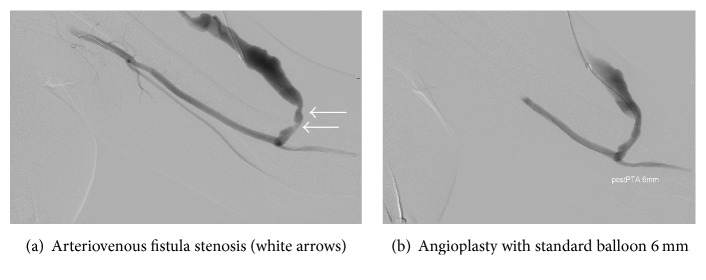# A Review of Percutaneous Transluminal Angioplasty in Hemodialysis Fistula

**DOI:** 10.1155/2018/1420136

**Published:** 2018-03-27

**Authors:** Ioannis Bountouris, Georgia Kritikou, Nikolaos Degermetzoglou, Konstantinos Ioannis Avgerinos

**Affiliations:** Department of Vascular Surgery, 251 General Hospital of Hellenic Air Force, Athens, Greece

## Abstract

The number of patients in dialysis increases every year. In this review, we will evaluate the role of percutaneous transluminal angioplasty (PTA) according to patency of arteriovenous fistula and grafts. The main indication of PΤΑ is stenosis > 50% or obstruction of the vascular lumen of an arteriovenous fistula and graft. It is usually performed under local anesthesia. The infection rate is as low as the number of complications. Fistula can be used in dialysis in the same day without the need for a central venous catheter. Primary patency is >50% in the first year while primary assisted patency is 80–90% in the same time period. Repeated PTA is as durable as the primary PTA. An early PTA carries a risk of new interventions. Cutting balloon can be used as a second-line method. Stents and covered stents are kept for the management of complications and central outflow venous stenosis. PTA is the treatment of choice for stenosis or obstruction of dialysis fistulas. Repeated PTA may be needed for better patency. Drug eluting balloon may become the future in PTA of dialysis fistula, but more trials are needed.

## 1. Introduction

Chronic kidney disease (CKD) is defined as damage of kidney or glomerular filtration rate (GFR) < 60 ml/min/1.73 m^2^, due to any cause, for at least three months [1]. Chronic kidney disease is accompanied by poor outcomes such as cardiovascular complications and premature death [2]. The last stage of CKD is kidney failure (GFR < 15 ml/min/1.73 m^2^) [1].

Common modalities for treatment of patients with kidney failure, before kidney transplantation, are hemodialysis (HD) and peritoneal dialysis (PD). A vascular access site which can be either an arteriovenous fistula (AVF) or arteriovenous graft (AVG) or venous catheter is required for the application of HD [3]. Arteriovenous (AV) fistulas are usually the first choice for vascular access in those undergoing HD, as they are more durable and have decreased risk for infection in comparison with AVGs. If U/S shows that vessels are not suitable for AVF, then an AVG is tried. The venous catheters are usually used for access until the time placement of AVF or AVG. Permanent venous catheters are the last access when a patient has lost all the possible sites for a AVF or AVG. The last decade new hybrid grafts, as Hemodialysis Reliable Outflow (HeRO) graft (Merit Medical Systems, Inc., USA), are also used in cases of central venous stenosis or occlusions.

A complication of AVFs and AVGs is significant stenosis (>50% of the lumen) or obstruction and is usually restored with percutaneous transluminal angioplasty (PTA) or surgical intervention [4]. In the present review, we summarize the role of PTA in the restoration of patency of stenotic AVFs and AVGs.

## 2. Angioplasty, Then and Now

Angioplasty for stenosis of AVFs was, for the first time, reported in 1981 [5]. The technique (“Grüntzig balloon catheter”) was feasible in three of the five patients of the study and showed encouraging results [5]. Since that time, there is much progress regarding PTA on AVFs and AVGs [6]. During last years, some researchers have been investigating the use of drug eluting balloons (DEB) which are balloons covered with drugs (mainly paclitaxel) possibly inhibiting restenosis [6–8]. These studies reflect the need of the implementation of a method or material that can offer the best possible patency with the least possible side effects for the vascular wall of the AVF/AVG.

In experimental animal studies, perivascular coverage of AVGs with paclitaxel, nitric oxide (NO), or dexamethasone was studied for its antistenotic action on graft [9]. The external wall of the vessel (adventitia) was considered as source of endothelial cells and the cause of vasoconstriction [9]. In another study, injected polymer with antiproliferative properties resulted in inhibition of neointimal hyperplasia of AV grafts [10].

## 3. Indications for Angioplasty of Vascular Access Sites

The basic indication for angioplasty of AVF or AVG in a HD patient is when there is stenosis > 50% of lumen's diameter which is accompanied by previous thrombosis, increased venous pressure during HD, worsening laboratory findings such as hyperkalemia and uremia, diminished murmur on auscultation of the vascular access, and finally drop of blood flow in color Doppler of the site [4].

## 4. Technique

Most vascular access procedures are performed with the use of topical anesthetics, but when central venous recanalization is needed along with angioplasty, general anesthesia may be implemented [11].

Angioplasty is usually preceded by a color U/S for the identification of the stenotic area. In cases of acute obstruction, angioplasty is performed after thrombolysis and angiography of the area of interest.

Depending on the stenosis site, the insertion of wires and catheters is performed according to the direction or opposite of the blood flow direction (Figure 1(a)) or both. The balloons that can be used in angioplasty are of three types: “standard” (Figure 1(b)), “high pressure,” or “cutting” [12]. Angioplasty is accompanied by the use of stent or stent graft [13]. While placing the stent or stent graft, the surgeon should consider a possible future stenosis and allow additional space for future new intervention with stent/stent graft [14].

Self-expanding stents are preferred because they have little risk of migration [15]. Their diameter has to be at least 1-2 mm greater than that of the biggest balloon's diameter [15].

The result of angioplasty is directly tested with intraoperative angiography and can be also clinically examined after the procedure [16].

## 5. Angioplasty versus Surgery

The choice for PTA or surgery for the treatment of stenosis of AVFs/AVGs depends on the experience of the vascular surgeon [17]. However, many centers around the world report an increased number of PTAs over surgery [18]. In any case, the target of both techniques has to be 50% for primary patency during the first 6-month period [17]. Angioplasty is a quick intervention with low risk of infection. There is no need for placement of permanent catheter and HD is feasible during the same day after intervention. In a retrospective study of 1987, the annual patency was 19.3% for surgical method while it was 31.3% for angioplasty [19]. However, many researchers believe in the superiority of surgical management with the placement of graft over PTA [19]. There is need for less reprocedures with surgical method but primary patency of the two methods is the same [20, 21]. According to Tordoir et al., surgical method is superior compared to PTA, in management of thrombosed AVFs but the two methods have same results in management of thrombosed synthetic AVGs [22]. In three studies, angioplasty is suggested as method of choice in management of AVF stenosis, while surgical method is suggested in case of PTA's failure [23–25].

## 6. Primary and Secondary Patency after PTA

Most studies report 6-month and even one-year primary patency of 50% [15, 18, 26–28]. However, there is need for repeated angioplasty because of the unavoidable hyperplasia of the vessel wall that is caused by the balloon use [29, 30]. In Bountouris et al. study, repeated PTA resulted in assisted primary patency of 85% and surgery resulted in secondary patency of 91%, at one year [18]. The patency of the new angioplasty is the same as that of the initial angioplasty, finding that is different from the opinion that surgical method should follow in case of restenosis three months after angioplasty [17]. Ayez et al. showed in their study that repeated PTAs result in secondary patency of 77.8%, at two years [31].

It is believed that an early performed angioplasty is vulnerable to restenosis and this increases the number of possible new angioplasties [18, 32, 33]. Interestingly, Manninen et al. showed that age of fistula Brescia Cimino at the time of first angioplasty does not affect the result and that the most important predicting factors for future restenosis are the site of stenosis and the existence of stenosis in the region of anastomosis or in a small diameter vessel [34]. In another study, it is reported that stenosis of length greater than 2 cm is also a predisposing factor for restenosis after angioplasty [35].

There are few studies in literature comparing balloons with stents. It is possible that the use of cutting balloons was associated with better patency in comparison with high-pressure balloons and standard PTA [36, 37].

## 7. Monitoring of Vascular Access Sites

There are studies supporting the need for monitoring of AVFs/AVGs with U/S every three months [38]. The same studies support the idea of preventive angioplasty in asymptomatic stenosis [38]. However, the appropriate cooperation between nephrologists, surgeons, and nursing stuff, when accompanied by increased surveillance, leads to favorable outcomes, too [38].

## 8. Complications of Angioplasty

A frequent complication of angioplasty is rupture which can be treated conservatively [39, 40]. In some cases, there is need for stent or covered stent placement [39, 40]. Blood transfusion is rarely needed [39, 40]. The use of cutting balloons is associated with increased risk of rupture [40]. The appropriate sizing and selection of the balloon can minimize the ruptures [41].

## 9. Novel Methods

The use of balloons covered with paclitaxel is safe and helps in decreasing the risk of restenosis of AVFs/AVGs [6–8, 42–46]. However, there are only few studies supporting this finding and further research is needed on the topic [47, 48]. New hybrid grafts like HeRO are also used when there is central venous stenosis or occlusion [49]. Even in such a material, PTA can be used as a bail-out procedure [50].

## 10. Conclusion

The increasing number of patients with renal dysfunction depending on hemodialysis creates a large number of people needing a procedure to keep their fistula open. PTA is the gold standard in a stenosis of more than 50% of the lumen of a hemodialysis AVF and AVG or even in occlusion. Primary patency is more than 50% in the first year and primary assisted patency is 80–90% in the same time period. Repeated PTAs have the same patency. An early PTA from the creation of a fistula has a high risk of restenosis.

Cutting balloons and stents are kept in the armamentarium for treating more complicated cases or occlusions. Drug eluting balloons are a novel method that is trying to decrease the trauma in the endothelium of the vascular wall of a fistula. More trials are needed to find out if this more expensive material can increase the patency of a fistula.

## Figures and Tables

**Figure 1:**